# *Ten-a* Affects the Fusion of Central Complex Primordia in *Drosophila*

**DOI:** 10.1371/journal.pone.0057129

**Published:** 2013-02-20

**Authors:** Xuebo Cheng, Huoqing Jiang, Weizhe Li, Hailong Lv, Zhefeng Gong, Li Liu

**Affiliations:** 1 State Key Laboratory of Brain and Cognitive Science, Institute of Biophysics, Chinese Academy of Sciences, Beijing, People's Republic of China; 2 University of the Chinese Academy of Sciences, Beijing, People's Republic of China; 3 School of Medicine, Zhejiang University, Hangzhou, Zhejiang, People's Republic of China; 4 Key Laboratory of Mental Health, Chinese Academy of Sciences, Beijing, People's Republic of China; University of Missouri, United States of America

## Abstract

The central complex of *Drosophila melanogaster* plays important functions in various behaviors, such as visual and olfactory memory, visual orientation, sleep, and movement control. However little is known about the genes regulating the development of the central complex. Here we report that a mutant gene affecting central complex morphology, *cbd* (*central brain defect*), was mapped to *ten-a*, a type II trans-membrane protein coding gene. Down-regulation of *ten-a* in pan-neural cells contributed to abnormal morphology of central complex. Over-expression of *ten-a* by *C767*-Gal4 was able to partially restore the abnormal central complex morphology in the *cbd* mutant. Tracking the development of FB primordia revealed that *C767*-Gal4 labeled interhemispheric junction that separated fan-shaped body precursors at larval stage withdrew to allow the fusion of the precursors. While the *C767*-Gal4 labeled structure did not withdraw properly and detached from FB primordia, the two fan-shaped body precursors failed to fuse in the *cbd* mutant. We propose that the withdrawal of *C767*-Gal4 labeled structure is related to the formation of the fan-shaped body. Our result revealed the function of *ten-a* in central brain development, and possible cellular mechanism underlying *Drosophila* fan-shaped body formation.

## Introduction

The central complex is an interconnected neuropil structure across and along the sagittal mid-section of the fly brain and includes the protocerebral bridge (PB), the fan-shaped body (FB), the paired nodule (NO), and the ellipsoid body (EB). It is involved in multi-modal behavioral control, such as locomotion [1]–[3], visual pattern memory [4], [5] and spatial orientation [6], [7]. The development of the central complex can be traced back to the larval stage [8], [9]. Lineage analysis has revealed the neurons that contribute to the central complex [8], [9], [10], but the molecular and cellular mechanism of central complex formation is not fully understood.

In the 1980s, Martin Heisenberg and coworkers generated a series of structural mutants, in which the morphology of adult central brain structures like mushroom bodies and the central complex were destroyed [1], [11], [12]. Among these mutants, *mbm* (*mushroom body miniature*), *ceb* (*central brain deranged*) and *nob* (*no-bridge*) have been identified. *mbm* was found to be a transcription factor, a nucleic acid-binding zinc finger protein [13], while *ceb* was reported to encode Neuroglian, a cell adhesion molecule that is crucial for axonal development, synapse formation and female receptivity [14]–[17]. As to *nob*, it interacted with *drl* at the interhemispheric junction to affect the formation of protocerebral bridge [18]. Another mutant type is *central body defect* (*cbd*), of which the most typical phenotype is that the fan-shaped body and the ellipsoid body are fragmented in the middle, or some fusion of the fan-shaped body and the ellipsoid body. So far, the molecular basis of most structural mutants is unclear.

Ten-a belongs to a large protein family, Teneurin, which contains an N-terminal intracellular domain, a single transmembrane domain, eight EGF-like domains, a 6-blade β-propeller TolB-like domain, and 26 YD repeats [19], [20]. From invertebrates to vertebrates, Teneurins function as signaling molecules at the cell surface as type II transmembrane receptors, while the intracellular domain cleaved from membrane works as a transcription regulator [21]–[23] and carboxyl terminus functions as a bioactive peptide [24]–[27]. The Teneurin family members are thought to be important for establishment and maintenance of neuronal connections, neurite outgrowth and axon guidance [20]. Recent reports showed that two *Drosophila* Teneurin members, Ten-a and Ten-m, are crucial for proper synaptic matching and the maintenance of neuromuscular junction [28], [29]. Although Teneurin may play a role in mammalian brain function [20], [30], detailed study is still largely lacking.

Here, we report that the *Drosophila* structural mutant gene *cbd*, the most typical phenotype of which is the fragmented fan-shaped body and ellipsoid body, is *ten-a*. The *cbd* mutation disrupts the formation of the FB, by preventing the merging of the two FB parts. This defect was rescued by over-expression of *ten-a* in a *C767*-Gal4 labeled structure which separated the FB parts but later disappeared to allow the merging of the two FB primordia. Our results might reveal the molecular and cellular mechanism of *Drosophila* central complex development.

## Materials and Methods

### Fly strains

Flies were cultured on standard cornmeal food at 25°C with a 12 h light : 12 h dark cycle at 60% humidity [31]. Wild-type flies Berlin (*WTB*) and *w^1118^* were used in our study. The *cbd* lines were generated by EMS mutagenesis of *WTB* [32]. The deficiency lines (*Df(1)ED7161, Df(1)ED7153, Df(1)KA10, Df(1)RC29*, *Df(1)ED7147*), and flyC31 (y^1^M{vas-int.Dm}ZH-2Aw*; M{3xP3-RFP.attP}ZH-86Fb) were obtained from the Bloomington *Drosophila* Stock Center (Indiana University). The *ten-a* RNAi line (*w^1118^; P{GD3330}v8322*) was obtained from the Vienna *Drosophila* RNAi Center (Vienna, Austria). NP6510 was obtained from *Drosophila* Genetic Resource Center (Kyoto Institute of Technology, Japan). The *ten-a* imprecise jump out line *ten-a^#900^* was a gift from Dr. Ron Wides.

### DNA Sequencing

To determine whether there was a mutation in genes between 11A5 and 11A7, we sequenced the coding regions of *CG1924, CG32655*, and *ten-a* in wild-type flies (*WTB*) and *cbd* flies. The genome fragments were PCR amplified using *Pfu* polymerase (Promega). The gene-specific primers were designed according to the FlyBase Release 5.45 genome sequence (www.flybase.org). The primers for amplification also were used for sequencing for genes *ten-a* and *CG32655*. For gene *CG1924*, besides primer30 for amplifying and sequencing, other sequencing primers (S1, S2) were used.

Primers for *ten-a*:

Primer1: 5′-GGATCGTGGGCATCGGCGGTG-3′ and 5′-TTTACAAATTAGTTGAC-3′;

Primer2: 5′-GGAAGTTGGGTTCCATAGCA-3′ and 5′-GGCATCTATTTCCAGCCTGA-3′;

Primer3: 5′-CCCAACTGAGCGAGGAAATA-3′ and 5′-ACAATGTGGAGGTTCCAACG-3′;

Primer4: 5′-CAACAGACTGTTAGGCAAGAGA-3′ and 5′-TTGCACGCTTTTTCCCTATC-3′;

Primer5: 5′-GATAGGGATTTCGACGCAGA-3′ and 5′-AAGTGCATCGAGTGCATTATTTA-3′; 

Primer6: 5′-TTCGAGTGCATCCCAAAAAT-3′ and 5′-CCCATATTCCGCATCTCCTA-3′;

Primer7: 5′-CCCACCCCCTTTTTGTTAAT-3′ and 5′-TTCTTAGCTGGCCGAAGTGT-3′;

Primer8: 5′-CGGCAGATAAGATGAAACAACA-3′ and 5′-GCCTCGTTGAACTCCTTCAG-3′; 

Primer9: 5′-GTGGCATAATGAATGGTGGA-3′ and 5′-CTGCAGCAGGGATACATTCA-3′;

Primer10: 5′- GATACGGCCAAACAGCATCT-3′; and 5′-CGGGATTCCCCGTTATATTC-3′

Primer11: 5′-AAAACCACCAAATGCTGACC-3′ and 5′-AGCTCGTGATTTCCAGTTCC-3′; 

Primer12: 5′-TAAATGCGCACAATGGAAAA-3′ and 5′-ACCGCAATGTTGCTGTTGTA-3′; 

Primer13: 5′-CAATTCGATTGCGTGTCAAG-3′ and 5′-GAATCCCTGCGCACTAAGAG-3′;

Primer14: 5′-GCAAAAACTCGAACGCAAAT-3′ and 5′-GCAAAAACTCGAACGCAAAT-3′; 

Primer15: 5′-ATTTCTCATGCCACCCACTC-3′ and 5′-TGGTAAATGAGGGGCACTTT-3′; 

Primer16: 5′-CTGTCACCTGAGACCGATGA-3′ and 5′-ATTGCAGTAATCCGGACAGG-3′; 

Primer17: 5′-GGAGTATCCGAGAACCGTCA-3′ and 5′-GGATCTTCATGTCCGAGGTG-3′; 

Primer18: 5′-CATGGCCATCACAATCACTG-3′ and 5′-TAGCAGCGAACCTAATCGAA-3′; 

Primer19: 5′-TCTAACACGCATTTCCCTCA-3′ and 5′-AAAATCCCACGAAAAACGTC-3′;

Primer20: 5′-CAGGTCAAATAGTGCGAATGC-3′ and 5′-CCCATGGTGACACTTTGATG-3′; 

Primer21: 5′-CCCTAGTGATTCATGGCGATA-3′ and 5′-GAAGTCCCATCGGTACTCCA-3′; 

Primer22: 5′-CTGCTCCAGACGATCCTACC-3′ and 5′-GCTCTTCTCTGGCATTTTGG-3′; 

Primer23: 5′-GCCCAGGACAGGATTGTAAA-3′ and 5′-CGGCAAAGTCCTCTGGATAG-3′;

Primer24: 5′-CGTGTTGCCGAGAGGATTAT-3′ and 5′-GCTGGTCCACTACCCACAGT-3′; 

Primer25: 5′-GCAGGACTCGTTCTTCTTCG-3′ and 5′-CTGTTCTTCGGTTTTCACTGC-3′; 

Primer26: 5′-CGCAGATCCACCGATCTAAT-3′ and 5′-GTTGCCGTTCAATTGGTTTT-3′;

Primer27: 5′-TTTATGGGAATGGGCGTATC-3′ and 5′-GCATTGAGCTGAGTTCGAGA-3′;

primers for *CG32655*

Primer28: 5′-GAGCGACTGAGGATTCCCTA-3′ and 5′-TGGGTCTTTCGCTAGTCGTT-3′; 

Primer29: 5′- CAGTGCTAGTTCCGTCGTGA-3′ and 5′-TGAAAAGGCTGGCTAGTTGG-3′;

primers for *CG1924*

Primer30: 5′-CCGGGAAAACTGTTGAAAAA-3′ and 5′-TATGAATGCCCGCTTACTCC-3′;

S1: 5′-TTCAAGTCGGAGAAGCCACT-3′

S2: 5′-ATCATCCGCAATCCCAACTA-3′

### Construction of transgenic flies UAS-*ten-a*

The first strand cDNA of *ten-a* was synthesized from fly head mRNA using the SuperScript III first-strand synthesis system (Invitrogen). PCR reactions were performed by *Pfu* polymerase (Promega) with 5′ primer 5′-GCAGTCAGATCTATGACTATGAAATCGATGAAG-3′ and 3′ primer 5′-ATTACTGACGTCGGTACCCTAACAGTCGGCTTCGCG-3′. BglII and KpnI adapters were added to the 5′ and 3′ primers, respectively. The 9042 bp product was inserted into the BglII and KpnI sites of pUASTattB (a gift from Konrad Basler) after the sequence was confirmed. The purified construct was introduced to FlyC31 strains.

### Immunohistochemistry

Flies were allowed to lay eggs on standard fly food for 30 min and brain dissection was performed at different time points of development: larvae, 48–72 h, 72–96 h and 96–120 h after egg laying; pupa, 0–2 h and 8–9 h after puparium formation; adult fly, 3∼5 days after eclosion. Immunostaining was performed as described previously [33] with modifications. Briefly, dissection was performed in a dish covered with cold PBS (phosphate-buffered saline, pH 7.4). The samples were fixed in freshly prepared paraformaldehyde (4% in PBS) for 3 h on ice. Brains were then washed in PBT (PBS+0.5% Triton X-100) for 3×15 min, followed incubation for 1 h in PNT (PBT+10% normal goat serum) at room temperature. Subsequently, rat monoclonal antibody to DN-cadherin (DSHB, DN-EX #8, dilution 1∶100) was used to track the development of the central complex in the larval and pupal stage. Nc82 antibody (DSHB; mAb nc82, dilution 1∶100) against Brp protein was the primary antibody used to stain the neuropil in adult flies. Rabbit anti-GFP antibody (Invitrogen Inc, dilution 1∶1000) was used to check the expression pattern of Gal4 lines. After incubating overnight at 4°C in primary antibody, samples were washed in PBT 3×15 min at room temperature, and then incubated overnight in secondary antibody. Goat anti-Rat antibody (TRITC-conjugated, Jackson Laboratories, dilution 1∶200), goat anti-Mouse antibody (TRITC-conjugated, Jackson Laboratories, dilution 1∶200), and goat anti-rabbit antibodies (FITC-conjugated, Invitrogen Inc, dilution 1∶200) were used as secondary antibodies. In the following day, after being rinsed in PBT 3×15 min, brains were mounted in Vectashield Fluorescent Mounting Media (Vector Laboratories, Burlingame) and observed.

### Imaging

Mounted brains were scanned with a confocal microscope (Leica TCS SP5). Each brain stack z-resolution was 0.8 µm or 1.0 µm for adult fly brains and 0.5 µm for larval or pupal brains, at pixel resolution of 1024×1024. The images were then processed with ImageJ (National Institutes of Health, *rsbweb.nih.gov/ij/*).

### Statistics

A two-tailed Fisher exact test was used to evaluate the efficiency of rescue experiments, and two sample *t*-test was carried out for the numbers of F1 neurons or F5 neurons. Statistical significance was assigned, *P<0.05, **P<0.01, ***P<0.001.

## Results

### *cbd* is mapped to *ten-a*

**Figure 1:**
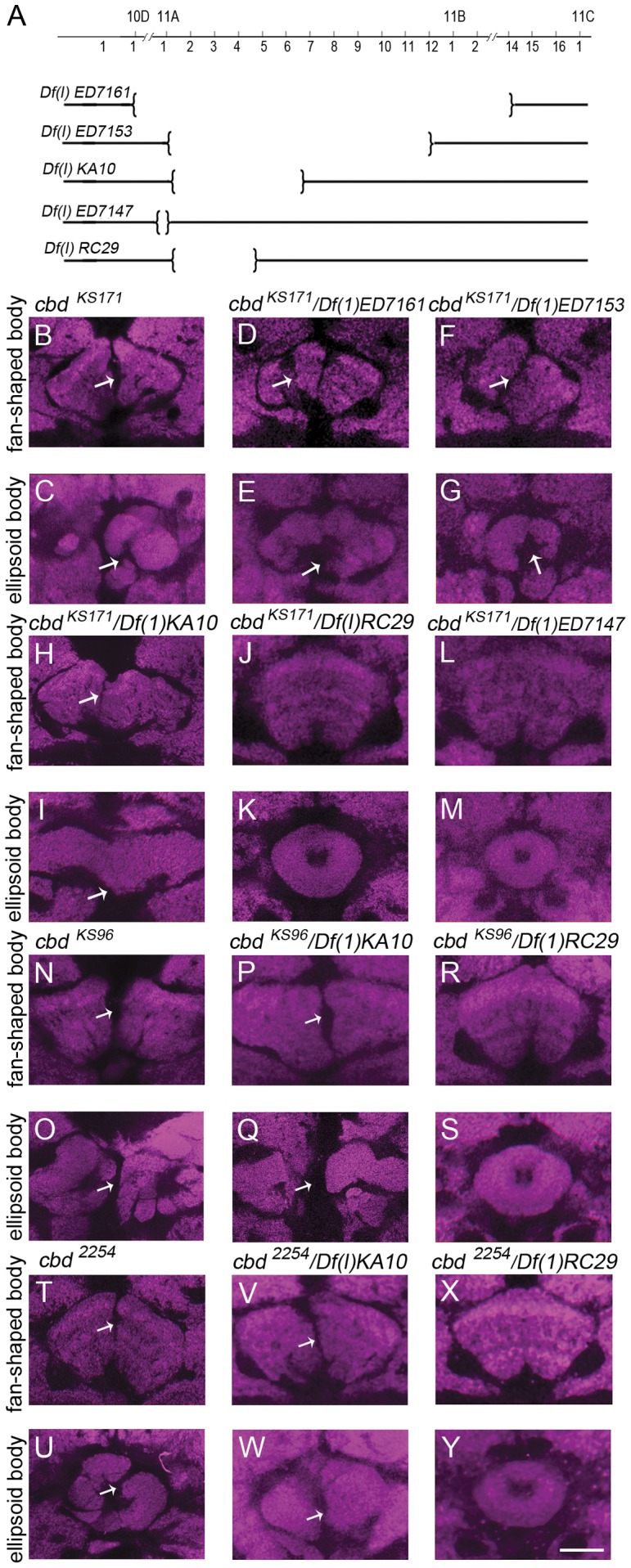
Morphology of the fan-shaped body (FB) and the ellipsoid body (EB) in the complementation test. (**A**) Schematic drawing of deleting regions of five deficiency lines on the X chromosome. (**B, C**) *cbd ^KS171^* mutant showed a defect in FB and EB. (**D–I**) complementation test *cbd ^KS171^* and *Df(1)ED7161 (D,E)* or *Df(1)ED7153 (F,G)* or *Df(1)KA10* (**H,I**) showed a defect in FB and EB. (**J–M**) complementation test *cbd ^KS171^* and *Df(1)RC29* or *Df(1)ED7147* showed a normal FB and EB. (**N, O**) *cbd ^KS96^* mutant showed a defect in FB and EB. (**P–S**) complementation test between *cbd ^KS96^* and *Df(1)KA10* showed a defect in FB (**P**) and EB (**Q**). *cbd ^KS96^* and *Df(1)RC29* showed a normal FB (**R**) and EB (**S**). (**T, U**) *cbd ^2254^* mutant showed a defect in FB and EB. (**V–Y**) complementation test between *cbd ^2254^* and *Df(1)KA10* showed a defect in FB (**V**) and EB (**W**) while that between *cbd ^2254^* and *Df(1)RC29* showed a normal FB (**X**) and EB (**Y**). Scale bar, 25 µm. *Arrows* indicate the central complex defect.

Although most structural mutants generated by Heisenberg and colleagues are still uncharacterized, some of them have been preliminarily mapped [13], [15], [18], [32]. The structural mutant gene *cbd*, which contributed to the disrupted fan-shaped body and ellipsoid body, was localized to 11A1–A7 on the X chromosome [1], [32]. To further localize *cbd*, we adopted a traditional method of deficiency mapping using complementation tests (Fig. 1). *cbd ^KS171^*, a representative *cbd* mutant, was crossed with a series of deficiency lines around the 11A region and the brain morphology of trans-heterozygous female offspring was checked. Three large deficiencies, *Df(1)ED7161, Df(1)ED7153, Df(1)KA10*, which respectively remove 10D1-11B14, 11A1-11B1 and 11A1–11A7, failed to restore the deranged central complex in *cbd ^KS171^* mutants, confirming that the *cbd* gene lies within 11A1–11A7 (Fig. 1B–I). Next we used deficiencies with a breakpoint between 11A1 and 11A7 for fine mapping. Both deficiency lines *Df(1)RC29* and *Df(1)ED7147*, that remove 11A1–11A5 and 10D6-11A1 respectively, could complement the *cbd ^KS171^* mutation, suggesting that *cbd* lies outside of 11A1–11A5 and locates in 11A5∼11A7 (Fig. 1J–M). The complementation tests for another two *cbd* mutants, *cbd ^KS96^* and *cbd ^2254^* confirmed that *cbd* was located in 11A5–11A7 (Fig. 1N–Y).

**Figure 2:**
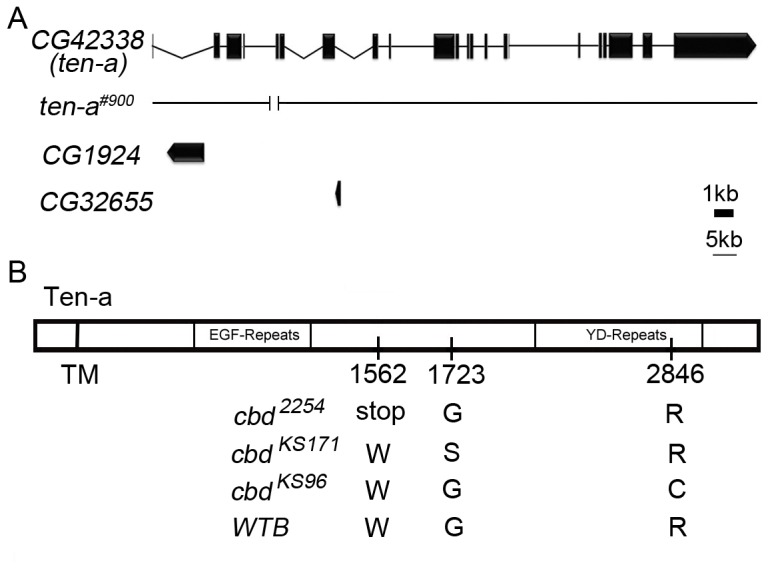
Schematic drawing of genomic region containing *ten-a*. (**A**) Schematic drawing of *CG42338* (*ten-a*), *CG1924*, *CG32655*, and *ten-a* deficiency line *ten-a^#900^*. (**B**) Schematic drawing of mutant site of Ten-a. In *cbd ^2254^*, Tryptophan at 1562 is changed to a premature stop codon. In *cbd ^KS171^*, Glycine at 1723 is changed to Serine. In *cbd ^KS96^*, Arginine at 2846 is changed to Cysteine.

We then checked the annotated genes in 11A5–11A7. According to FlyBase annotation release 5.45, there are three genes in this region: *CG1924*, *CG32655* and *CG42338*. We sequenced the coding DNA region of these three genes in the three *cbd* mutant lines *cbd ^KS171^*, *cbd ^2254^* and *cbd ^KS96^*, and found no sequence change in *CG1924* and *CG32655* that could lead to changes in amino acids or splicing sites. Interestingly, we found significant DNA sequence changes in the coding region of *CG42338*, also named *ten-a* (Fig. 2A). For convenience of illustration, the mutation points were assigned to the transcript RE, which encodes the largest protein isoform (3378 aa) according to the FlyBase annotation. In *cbd ^2254^*, a TGG codon (Tryptophan, 1562) was changed to TGA (a premature stop codon). In *cbd ^KS171^*, a GGC codon was changed to AGC (Glycine in 1723 changed to Serine). In *cbd ^KS96^*, the CGT codon was changed to TGT (Arginine 2846 changed to Cysteine) (Fig. 2B, S1). It is worth noting that all three amino acid changes occur in the conserved domains of the Ten-a protein (Fig. S2). Besides these three mutations, there are several amino acid residues that are same in the three *cbd* lines but different from control flies (*WTB*), and all are in non-conserved domain of Teneurin (Fig. S3). All of these data strongly hint that *cbd* is *ten-a*.

### *ten-a* is required for normal central complex morphology

**Figure 3:**
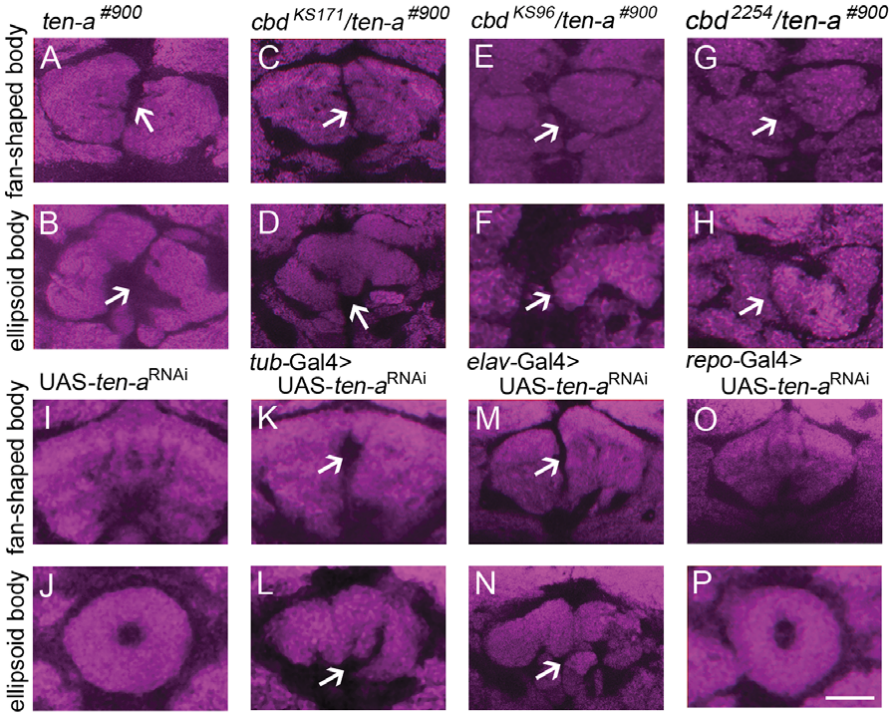
*ten-a* deficiency line *ten-a^#900^* and down-regulated *ten-a* expression by RNAi caused FB and EB defects. (**A, B**) *ten-a^#900^* showed a FB and EB defect. (**C–H**) *cbd ^KS171^*, *cbd ^2254^* and *cbd ^KS96^* could not complement the FB and EB defect of *ten-a^#900^*. (**I–L**) Both the FB and the EB were destroyed when *ten-a* was down-regulated by driving expression of UAS-*ten-a* ^RNAi^ with *tub*-Gal4. (**M–N**) Both the FB and the EB were destroyed when *ten-a* was down-regulated by driving expression of UAS-*ten-a* ^RNAi^ with pan-neuronal *elav*-Gal4. (**O–P**) Both the FB and the EB were normal when *ten-a* was down-regulated by driving expression of UAS-*ten-a* ^RNAi^ with pan-glial *repo*-Gal4. Scale bar, 25 µm. *Arrows* indicate the central complex defect.

Since *cbd* is likely *ten-a*, we wondered if manipulating *ten-a* gene expression would affect morphology of the fly central complex, as *cbd* mutations do. We first made use of the *ten-a* deficiency line *ten-a^#900^* which was generated by P element imprecise excision in *ten-a*. In *ten-a^#900^*, a 2219 bp region covering two *ten-a* exons was deleted (Fig. 2A). *ten-a^#900^* is homozygous viable, but the central complex morphology is disrupted (Fig. 3A, B). Complementation tests between *ten-a^#900^* and *cbd* mutants, *cbd ^KS171^*, *cbd ^KS96^* and *cbd ^2254^* showed that *ten-a^#900^* was unable to complement the *cbd* mutant based on brain morphology (Fig. 3C–H). Thus, *ten-a^#900^* is another *cbd* mutant allele. However, the question still remained that there might exist some other common mutation outside of *ten-a* in these mutant lines that generated the abnormal brain phenotype. We then specifically down-regulated *ten-a* expression by driving expression of UAS-*ten-a* ^RNAi^ with *tub*-Gal4. We noticed that morphology of both FB and EB was destroyed comparing with control flies (Fig. 3I–L). We further asked where *ten-a* functioned to affect fly brain morphology. By driving UAS-*ten-a* ^RNAi^ with pan-neuronal *elav*-Gal4 and pan-glial *repo*-Gal4, we found distinct results: down-regulation of *ten-a* in neurons produced strong brain derangement, while down-regulation of *ten-a* in glial cells had no effect on fly brain morphology (Fig. 3M–P). It suggests that *ten-a* is required in neurons for normal central complex morphology.

### Over-expression of *ten-a* restored normal brain morphology in *cbd* mutant

**Figure 4:**
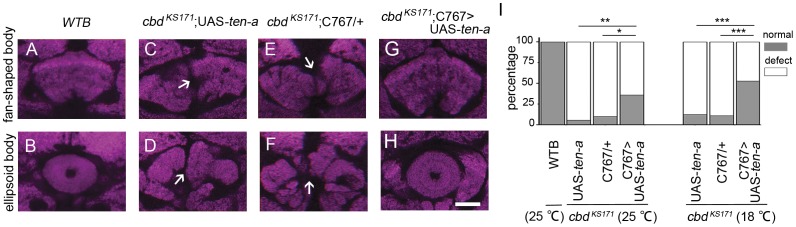
The *cbd* mutant phenotype could be significantly rescued when *ten-a* was driven by *C767*-Gal4. (**A, B**) *WTB* showed normal FB and EB. (**C–F**) Both UAS-*ten-a* and *C767*-Gal4 in the *cbd ^KS171^* background showed defect in FB and EB. (**G, H**) FB and EB were restored to normal when UAS-*ten-a* was driven by *C767*-Gal4. (**I**) Percentage of normal FB and EB in controls (5.6% for *cbd ^KS171^*;UAS-*ten-a*, n = 36; 10% for *cbd ^KS171^*;*C767*-Gal4/+, n = 30) and in flies with UAS-*ten-a* driven by *C767*-Gal4 (36%, n = 25). Two tailed Fisher exact test. *P<0.05, **P<0.01, ***P<0.001. When *ten-a* over-expressing flies were kept at 18°C, 47% (n = 32) of flies showed a normal FB and EB, much higher than control flies (12.5% for *cbd ^KS171^*;UAS-*ten-a*, n = 24; 11.1% for *cbd ^KS171^*;*C767*-Gal4/+, n = 18). Scale bar, 25 µm. *Arrows* indicate the central complex defect.

Next, we undertook fine mapping of the cells in which Ten-a functions to affect the central complex morphology by attempting to rescue the *cbd* mutant phenotype via over-expressing *ten*-a with screening various Gal4 lines. During the screening, pan-neuronal Gal4 line, glia-specific Gal4 line, and some region specific Gal4 lines were chosen for rescue experiments. Finally only *C767*-Gal4, which labeled the EB as well as the median bundle in adult central brain, significantly rescued the *cbd* mutant phenotype (Fig. 4). Since the phenotype is the cleavage of FB, it is reasonable to postulate that it is the *C767*-Gal4 labeled structure at midline but not at other regions that affected the FB morphology. Interestingly, the percentage of rescue was a little higher when flies were cultured at a constant temperature of 18°C comparing with flies cultured at a constant temperature of 25°C (Fig. 4I). Thus, Ten-a in *C767*-Gal4 labeled cells contributed to normal central complex formation.

### Defective FB in *cbd* mutants is caused by the failure of FB merging

**Figure 5:**
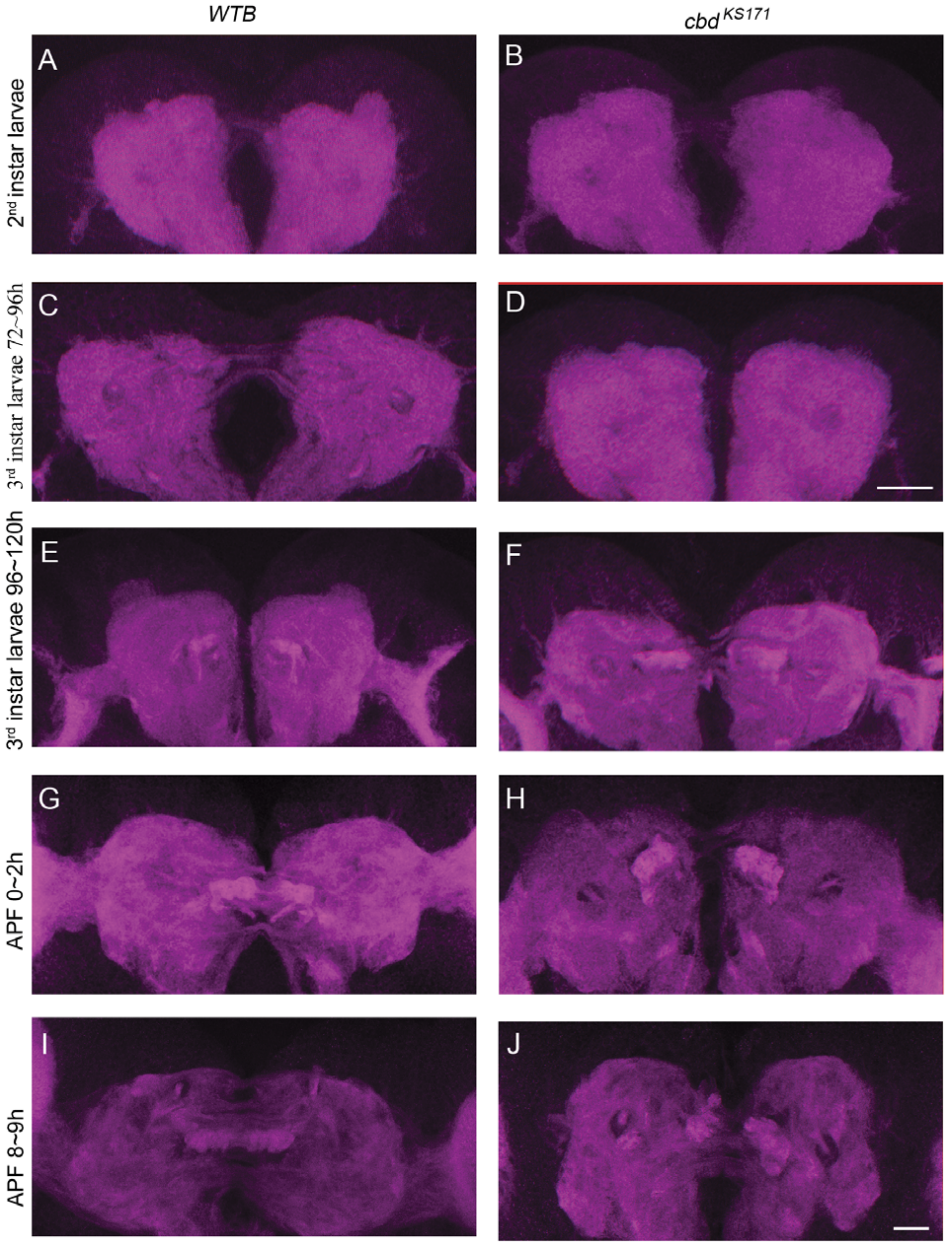
The development of the FB in both *WTB* and *cbd ^KS171^* from 2^nd^ instar larva to pupa. (**A–D**) DN-cadherin signal in the commissure in 2^nd^ instar larval brain of *WTB* (**A**) or *cbd ^KS171^* mutant (**B**), and in early 3^rd^ instar larval brain of *WTB* (**C**) or *cbd ^KS171^* mutant (**D**). Scale bar in (**D**) equals 25 µm and applies to (**A–D**). (**E–J**) FB precursors strongly labeled by DN-cadherin in the commissure in a late 3^rd^ instar larval brain from *WTB* (**E**) or a *cbd ^KS171^* mutant (**F**), and in a 0–2 h APF pupal brain from *WTB* (**G**) or a *cbd ^KS171^* mutant (**H**), and in an 8–9 h APF pupal brain from *WTB* (**I**) or a *cbd ^KS171^* mutant (**J**). Scale bar in (**J**) equals 25 µm and applies to (**E–J**).

We then asked how *ten-a* mutation led to the deranged central complex morphology. Theoretically, the deranged FB in *cbd* mutants could result from either abnormal development, or from degeneration of a normal mature FB. We tracked the whole developmental process of the FB in both wild-type and *cbd* mutants from 2^nd^ instar larva to pupa (Fig. 5), when the FB is supposed to form [8]. We could see several thin commissural axon tracts which was supposed as supraesophageal commissure [34] in 2^nd^ and early 3^rd^ instar larval brains in both wild-type and *cbd* mutants (Fig. 5A–D), which suggests that *ten-a* mutation doesn't affect the midline crossing of this structure, at least in these stages. In late 3^rd^ instar larval brains, we found immature FB stained by DN-cadherin anterior to the supraesophageal commissure on the two sides of the midline (Fig. 5E, F). The FB precursors expand and merge at the midline by 8–9 h after pupa formation (APF) in wild-type flies (Fig. 5G, I). In *cbd ^KS171^* flies, the similar event happens until 0∼2 h APF. However, in *cbd* mutants the two strong DN-cadherin labeled precursors were unable to merge at 8∼9 h APF (Fig. 5H, J), even at later time points that we have checked (data not shown). Thus, the broken FB in *cbd* mutants was likely caused by a developmental defect in FB formation, but not by neuronal degeneration of the formed FB.

### *C767*-Gal4 labeled structure at interhemispheric junction might be required for FB formation

**Figure 6:**
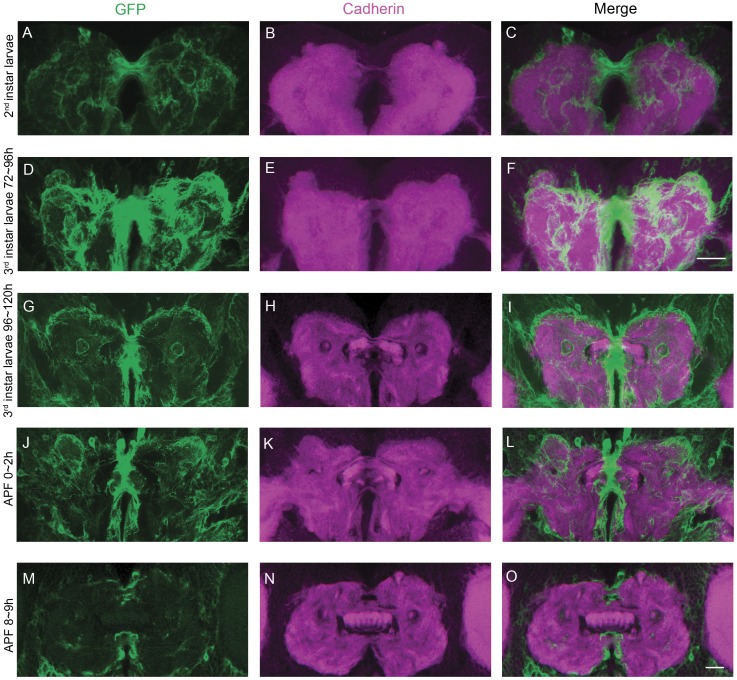
The expression pattern of *C767*-Gal4 and DN-cadherin signal during FB formation in wild type flies. (**A–C**), The expression pattern of *C767*-Gal4 (green) and DN-cadherin signal (magenta) in a 2^nd^ instar larval brain. (**D–F**) The expression pattern of *C767*-Gal4 (green) and DN-cadherin signal (magenta) in an early 3^rd^ instar larval brain. Scale bar in (**F**) equals 25 µm and applies to (**A–F**). (**G–I**) The expression pattern of *C767*-Gal4 (green) and DN-cadherin signal (magenta) in a late 3^rd^ instar larval brain. (**J–L**) The expression pattern of *C767*-Gal4 (green) and DN-cadherin signal (magenta) in a pupal brain at 0–2 h APF. (**M–O**) The expression pattern of *C767*-Gal4 (green) and DN-cadherin signal (magenta) in a pupal brain at 8–9 h APF. Scale bar in (**O**) equals 25 µm and applies to (**G–O**).

We then asked exactly how *ten-a* affected the process of FB development. As *cbd* mutant phenotype can be partially rescued by over-expressing *ten-a* in *C767*-Gal4 labeled structure, we supposed that the labeled cells are closely related to the event. The expression pattern of *C767*-Gal4 in the region of larval FB precursors was tracked during the process of FB formation in both wild-type flies and *cbd* mutants. At the 2^nd^ instar larval stage, *C767*-Gal4 labeled universally in the central brain, but was significantly strong in midline regions joining the two hemispheres (Fig. 6A–C). It is worth noting that the two DN-cadherin stained FB patches were separated by a *C767*-Gal4 labeled structure at interhemispheric junction (Fig. 6G, J, M). Later in the 3^rd^ instar larval stage, the interhemispheric junction was narrowed while the FB precursors expanded and invaded medially. In wild type background, the interhemispheric junction and the FB precursors are still tightly connected as if the withdrawal of *C767*-Gal4 labeled structure at interhemispheric junction was accompanied with the extending of the DN-cadherin signal (Fig. 6G). By the pupal stage, the *C767*-Gal4 labeled midline structure further shrank and disappeared completely to allow complete merging of the two FB primordium parts (Fig. 6J, M). Judging from the concurrency between the morphological changes in *C767*-Gal4 stained structure and DN-cadherin labeled FB primordia, we postulated that the merging of FB primordia was influenced by the *C767*-Gal4 labeled midline junction.

**Figure 7:**
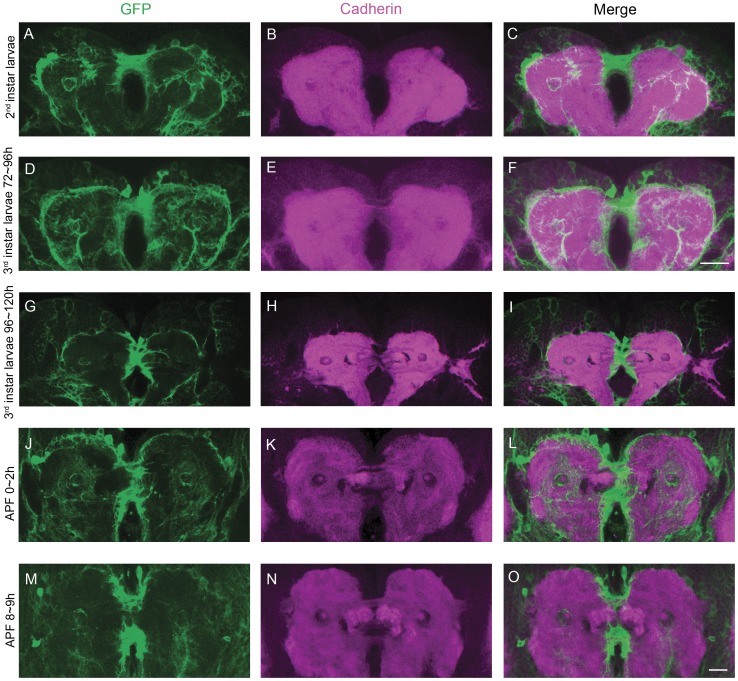
The expression pattern of *C767*-Gal4 and DN-cadherin signal during FB formation in *cbd ^KS171^* mutants. (**A–C**) 2^nd^ instar larval brain. (**D–F**) early 3^rd^ instar larval brain. Scale bar in (**F**) equals 25 µm and applies to (**A–F**). (**G–I**) late 3^rd^ instar larval brain. (**J–L**) pupal brain 0–2 h APF. (**M–O**) pupal brain 8–9 h APF. Scale bar in (**O**) equals 25 µm and applies to (**G–O**).

To confirm this hypothesis, we checked the development of *C767*-Gal4 labeled cells and FB morphology in *cbd ^KS171^* mutant (Fig. 7). In *cbd ^KS171^* mutant, the *C767*-Gal4 labeled midline structure that separated the two FB patches persisted during 0–2 h APF. But the structure is detached from the precursors and failed to guide the merging of FB parts and consequently the formation of a normal FB (Fig. 7). Based on this morphological observation, as well as the rescue results, we concluded that the Ten-a molecule in the *C767*-Gal4 labeled structure affected merging of FB primordia.

### Defective FB in *cbd* mutants is not caused by abnormal axonal projections

**Figure 8:**
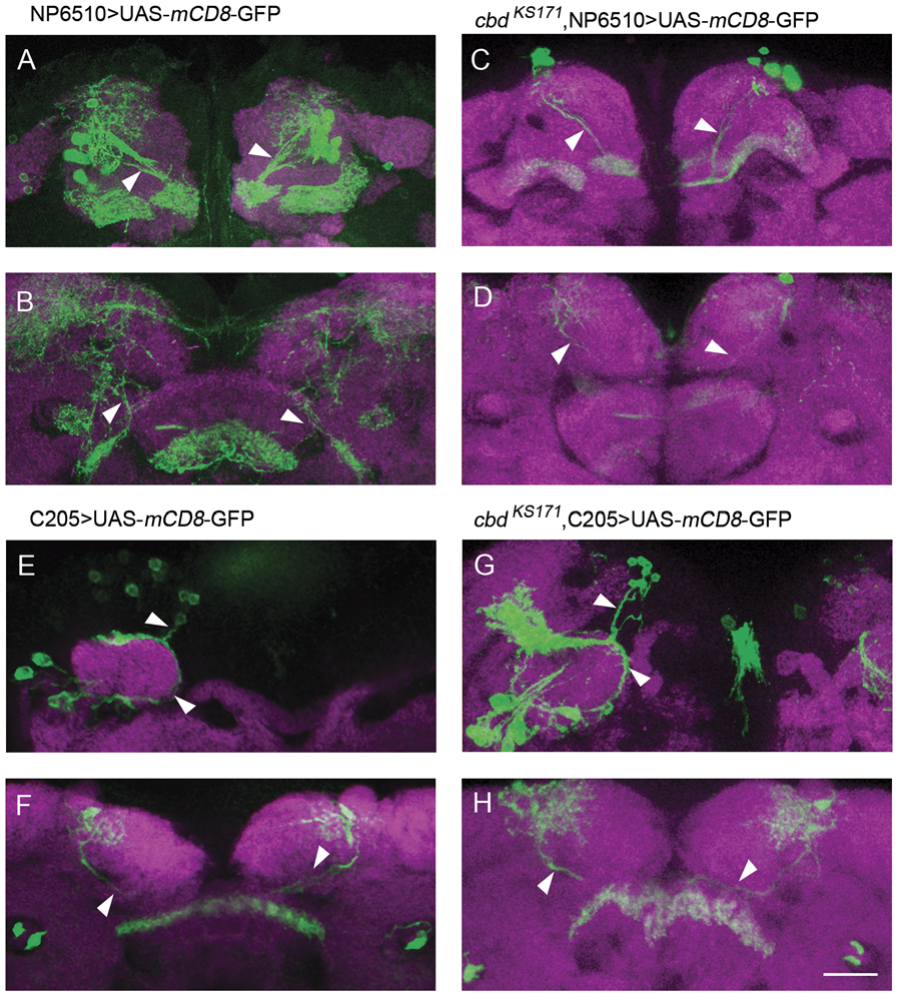
The projection tracts of both F1 neurons and F5 neurons appeared to be normal in *cbd ^KS171^* mutants. (**A–D**) Neural projection and arborizations of F1 neurons in wild type flies and *cbd ^KS171^* mutants. (**E–H**) Neural projection and arborizations of F5 neurons in wild type flies and *cbd ^KS171^* mutants. Scale bar, 25 µm. *Arrowheads* indicate the normal neural projection.

Since the deficiency is not due to the degeneration, we also wondered if the failure in FB part merging in *cbd* mutants was due to improper generation or specification of FB neurons. We counted the neurons labeled by *NP6510*-Gal4 and *C205*-Gal4 in both *cbd ^KS171^* mutant and wild type adult flies. The somata of F1 neurons were located anterior to the antennal lobes and the somata of F5 neurons were around the calyxes of mushroom bodies. The numbers of neurons varied even in each hemisphere in the same brain and there was no significant difference between wild type and *cbd ^KS171^* flies (Fig. 8A, C, E, G, Fig. S4). We also wondered if the abnormal morphology of FB was caused by abnormal axonal projections of FB neurons. The morphology of F1 neurons labeled by *NP6510*-Gal4 and F5 neurons labeled by *C205*-Gal4 were checked in adult *cbd* mutant flies. As shown in Figure 8, FB in *cbd ^KS171^* mutants were fragmented into pieces (Fig. 8D, H), but the projection tracts of both F1 neurons and F5 neurons appeared to be normal in *cbd ^KS171^* mutants (Fig. 8B, F). Thus, the mutant FB phenotype in *cbd* mutants is not caused by abnormalities in neuronal projection. Rather, the FB defect in *cbd* mutants was caused by abnormalities in growth of the FB midline-crossing arborizations, but terminal arborizations in the non-cleft region could still form in *cbd* mutants.

## Discussion

In this study, we found that the *Drosophila* central brain morphological mutant *cbd* is actually *ten-a*, a member of the teneurin family. Ten-a is required for fusion of the fan-shaped body precursors, before the formation of the complete normal FB. Mutation in *ten-a* leads to the failure of the two FB precursors to merge and consequently to the deranged fan-shaped body in adult flies.

Aside from the FB morphological defect itself, *ten-a* mutation might cause other abnormalities that contribute to the morphological defect. For example, Ten-a might affect the projections and contra-lateral crossing of FB neurons resulting from lineages of FBP1 and FBP2, which contribute to two staves of the fan-shaped body [10], consequently led to a cleaved fan-shaped body. Nevertheless, the generation and projection of large field ExF*l* neurons labeled by *NP6510*-Gal4 or *C205*-Gal4 are not affected when *ten-a* mutated (Fig. 8), which suggested that *ten-a* mainly produce the morphological defect by exerting its effect on FBP1 and FBP2 neuron arborizations. Actually, based on the morphological observation in *cbd ^KS171^* flies and the rescue results, we postulated that the interhemispheric structure *C767*-Gal4 labeled was related to FB primordial fusion. But it seemed to have no effect on axonal projections and terminal arborization of F1 and F5 neurons.

*ten-a* knockdown results showed that the neuronal *ten-a* was required for the central complex formation. Further rescue experiments suggested that neither neurons nor glial cells alone were sufficient for normal central complex formation. After screening, one Gal4 line was found finally. *C767*-Gal4 could be used to rescue the *cbd* mutant phenotype significantly. To identify the cell types labeled by *C767*-Gal4, we used neuron specific marker ELAV or glial cell specific marker REPO to co-stain with *C767*-Gal4 labeling cells. The results showed that nlsGFP driven by *C767*-Gal4 was co-localized with both neuronal and glial markers from larval to early pupal stages (Fig. S5). Since previous studies showed some adhesion molecules were expressed both in neurons and glia for mediating the fasciculation of axon bundles, axon guidance or targeting [35], we suggested that the rescue results by *C767*-Gal4 might just attribute to that the Gal4 expressed both in neurons and glial cells. That is to say, only when *ten-a* functions in certain neurons and glial cells together, the FB precursors could merge normally. However, we could not identify which neurons and glial cells were required for the partial rescue from current results. To solve this problem, more Gal4 lines which can rescue the *cbd* mutant phenotype are needed. Then, dependent on the expression patterns of these Gal4 lines, the neurons and glial cells which *ten-a* functions in may be identified.

If Ten-a functions in *C767*-Gal4 labeled cells to influence the merging of FB primordia, what is its working partner for the arborization of FB neurons? As a *Drosophila* homolog of vertebrate Teneurin, Ten-a has been reported to be involved in embryo development, especially in the central nervous system. Ten-a, as well as its homologue Ten-m, was recently found to be required for synaptic matching between olfactory receptor neurons and corresponding projection neurons [28]. Ten-a and Ten-m were also important for establishing the correct connection in the larval neuromuscular junction [29], [36]. In our work, lack of normal Ten-a function led to failure in merging of FB precursors. It is possible to assume that Ten-a itself mediates homophilic interaction between neurons and glial cells to regulate the fusion of the central complex, such as Nrg, which is expressed on both neurons and glial cells and interacts to control axonal sprouting and dendrite branching [37]. Meanwhile, Ten-a may interact with other molecules such as Ten-m, or other membrane proteins that function in heterophilic way at the cell surface. Further molecular and cellular experiments are needed to elaborate this important issue.

Vertebrate Teneurins have been suggested to be related to mental diseases and our discovery of Ten-a function in *Drosophila* brain development seems to support the hypothesis. Neuroglian (Nrg), whose vertebrate homologue L1-CAM has been implicated in neurological disorders [38], [39], is also required for development of normal brain morphology in *Drosophila* [40], [41]. Considering that both Nrg and Ten-a are type-II transmembrane proteins with extracellular EGF repeats and also function in glial cells for brain development [40], it is possible that Teneurins in vertebrates also affect brain development, and probably synapse formation, as vertebrate Nrg does.

In summary, our work elucidates the function of *ten-a* in development of the *Drosophila* central brain, and the cellular mechanism underlying FB formation.

## Supporting Information

Figure S1 **Sequence traces show the mutation in** ***cbd*** **lines.** (**A**) Sequence trace shows the nucleotide change of G in control flies to A in *cbd^2254^* leading to the nonsense mutation (W1562*). (**B**) Sequence trace in *cbd ^KS171^* shows G to A nucleotide change, leading to missense mutation (G1723S). (**C**) Sequence trace in *cbd ^KS96^* shows C to T nucleotide change, leading to missense mutation (R2846C). The *underlines* indicate the base substitution position.
(TIF)

Figure S2 **Conservation analysis of amino acids which were mutated in** ***cbd^2254^, cbd ^KS171^, cbd ^KS96^*** **, respectively.** Multiple-sequence alignment for Teneurin homologues surrounding the coding changes (*boxed*) was done by MegAlign program. We found the three regions all are with high conservation (*Cyan*), especially the changed sites, W1562, G1723, R2846, which can be found in all 20 homologues.
(TIF)

Figure S3 **Conservation analysis of amino acids that were seen in all three** ***cbd*** **lines, but not in control flies (** ***WTB*** **).** (**A**) Schematic drawing of sites that are different between *cbd* and control flies. At position 168, P in *WTB* was changed to A in all three *cbd* lines. D328 was changed to G328. I691 was changed to M691. L3372 was changed to F3372. (**B**) Conservation analysis of the sites. From the alignment results, we can see these sites are in a region with low conservation (*Cyan*) and the changed sites (*boxed*) are not appeared in other homologues.
(TIF)

Figure S4 **Average numbers of F1 and F5 neurons in control flies (** ***C205*** **-Gal4>UAS-GFP and** ***NP6510*** **-Gal4>UAS-GFP) and** ***cbd ^KS171^*** **mutant flies.** No significant difference of neuron numbers was observed between control flies (light grey) and *cbd ^KS171^* (dark grey), either for *NP6510*-Gal4 labeled F1 neurons (left) or for *C205*-Gal4 labeled F5 neurons (right). Two sample *t*-test, error bars represent the s.e.m.; n.s., not significant.
(TIF)

Figure S5 ***C767*** **-Gal4 labels both neurons and glial cells from the 3^rd^ instar larval to early pupal stage.** For easy illustration, multiple middle z-axis slices were stacked. GFP-labeled cell bodies (green) driven by *C767*-Gal4 co-localized with neurons (arrowheads) with a neural specific marker, ELAV, stained by anti-ELAV antibody (magenta) in 3^rd^ instar larval brain (**A**), pupal brain 0∼2 h APF (**B**), and pupal brain 8∼9 h APF (**C**). GFP-labeled cell bodies driven by *C767*-Gal4 co-localized with glial cells (arrowheads) with a glial specific marker, REPO, stained by anti-REPO antibody (blue) in 3^rd^ instar larval brain (**D**), pupal brain 0∼2 h APF (**E**), and pupal brain 8∼9 h APF (**F**). Schematics of distributions of neurons and glial cells in whole brains of 3^rd^ instar larva (**G**), pupa 0∼2 h APF (**H**), and pupa 8∼9 h APF (**I**). Scale bars, 25 µm.
(TIF)
