# Supplementary figures and images for: Ten-a Affects the Fusion of Central Complex Primordia in Drosophila
Source: PLoS One. 2013 Feb 20;8(2):e57129. doi: 10.1371/journal.pone.0057129 (PMC3577759; doi:10.1371/journal.pone.0057129)

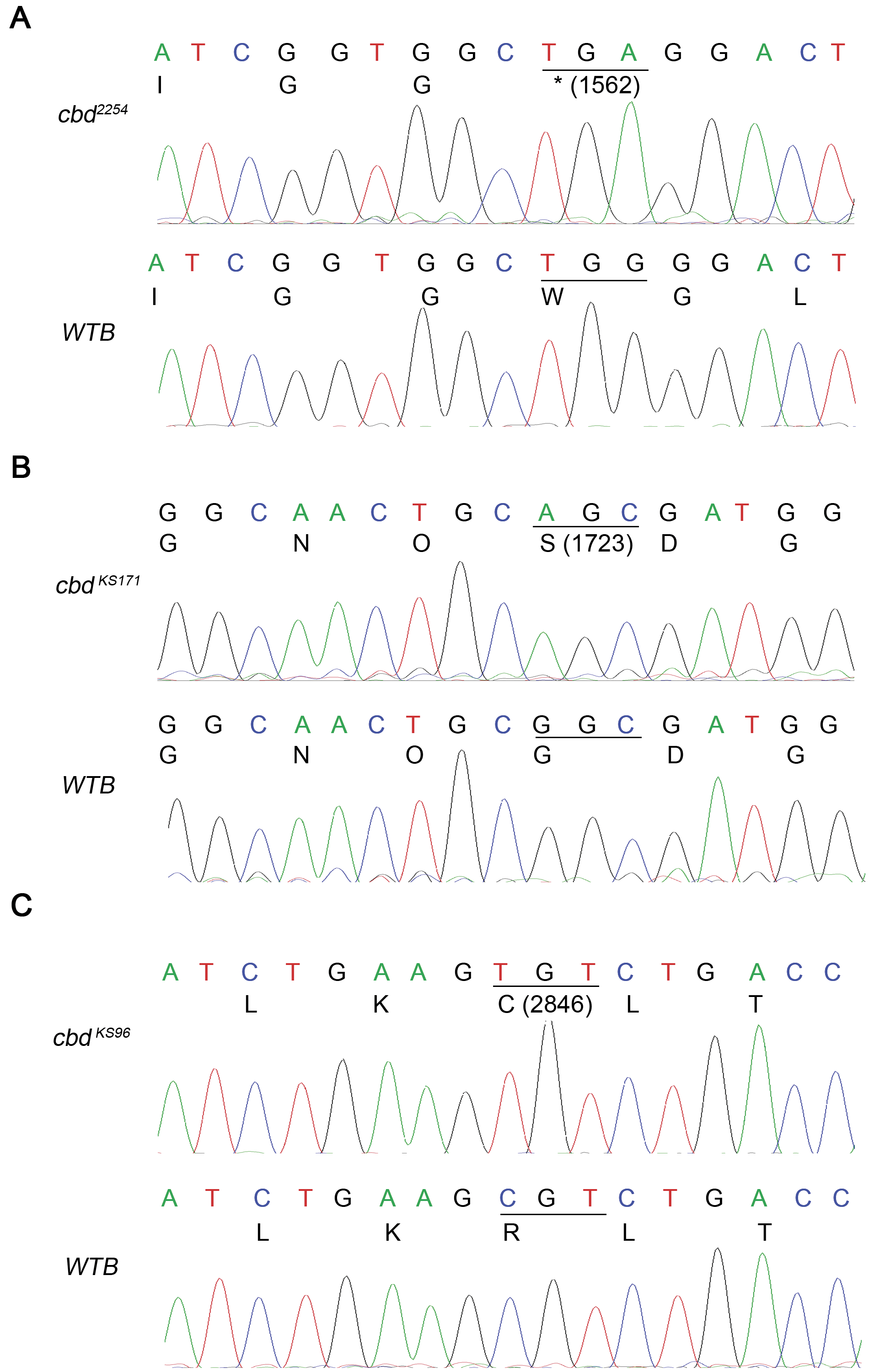

Supplement: Figure S1 — Sequence traces show the mutation in cbd lines. (A) Sequence trace shows the nucleotide change of G in control flies to A in cbd2254 leading to the nonsense mutation (W1562*). (B) Sequence trace in cbd KS171 shows G to A nucleotide change, leading to missense mutation (G1723S). (C) Sequence trace in cbd KS96 shows C to T nucleotide change, leading to missense mutation (R2846C). The underlines indicate the base substitution position. (TIF) [file pone.0057129.s001.tif]

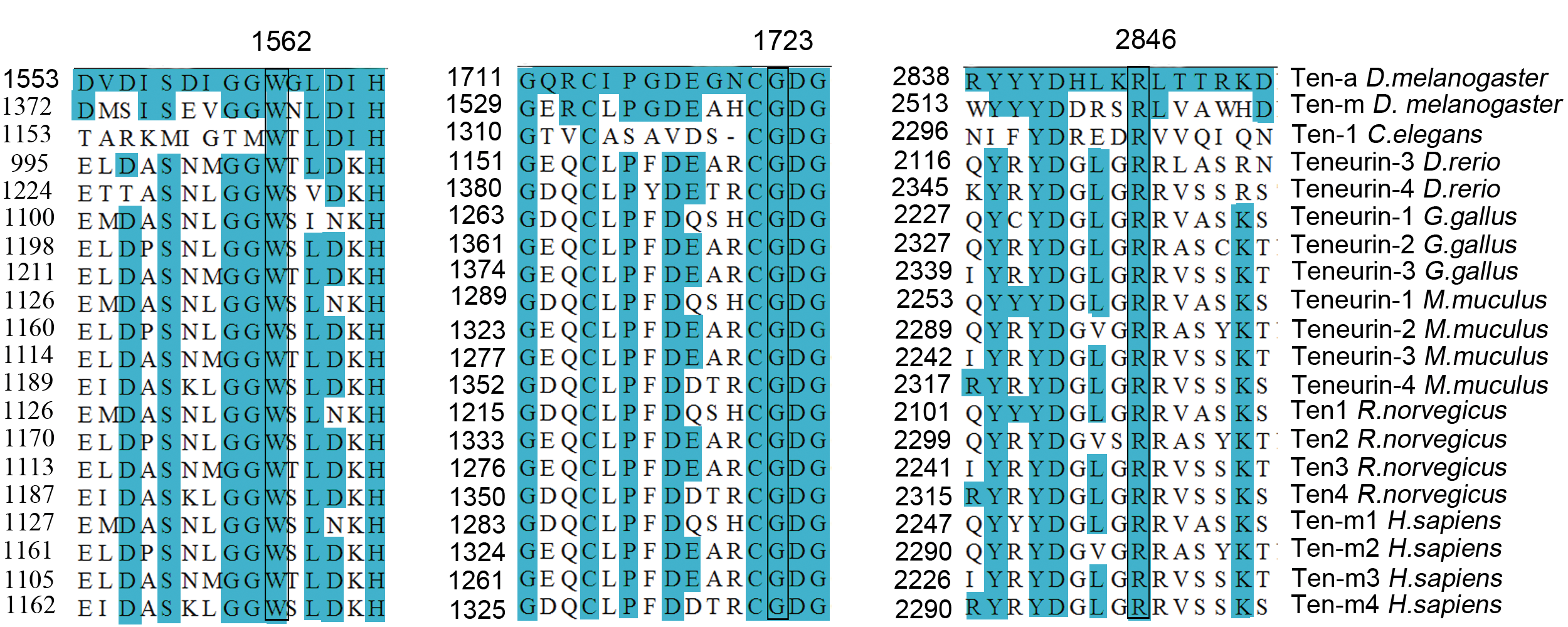

Supplement: Figure S2 — Conservation analysis of amino acids which were mutated in cbd2254, cbd KS171, cbd KS96 , respectively. Multiple-sequence alignment for Teneurin homologues surrounding the coding changes (boxed) was done by MegAlign program. We found the three regions all are with high conservation (Cyan), especially the changed sites, W1562, G1723, R2846, which can be found in all 20 homologues. (TIF) [file pone.0057129.s002.tif]

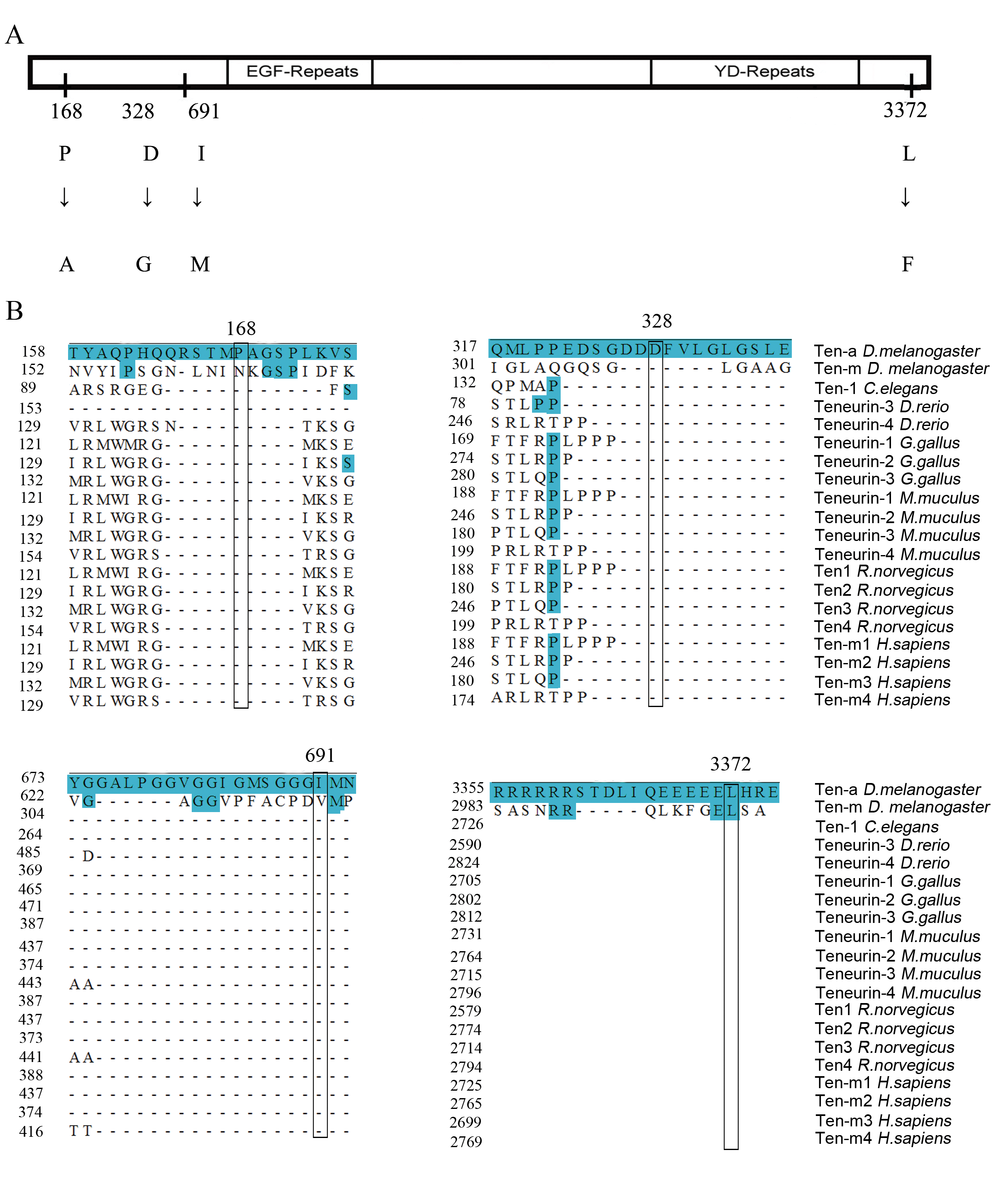

Supplement: Figure S3 — Conservation analysis of amino acids that were seen in all three cbd lines, but not in control flies ( WTB ). (A) Schematic drawing of sites that are different between cbd and control flies. At position 168, P in WTB was changed to A in all three cbd lines. D328 was changed to G328. I691 was changed to M691. L3372 was changed to F3372. (B) Conservation analysis of the sites. From the alignment results, we can see these sites are in a region with low conservation (Cyan) and the changed sites (boxed) are not appeared in other homologues. (TIF) [file pone.0057129.s003.tif]

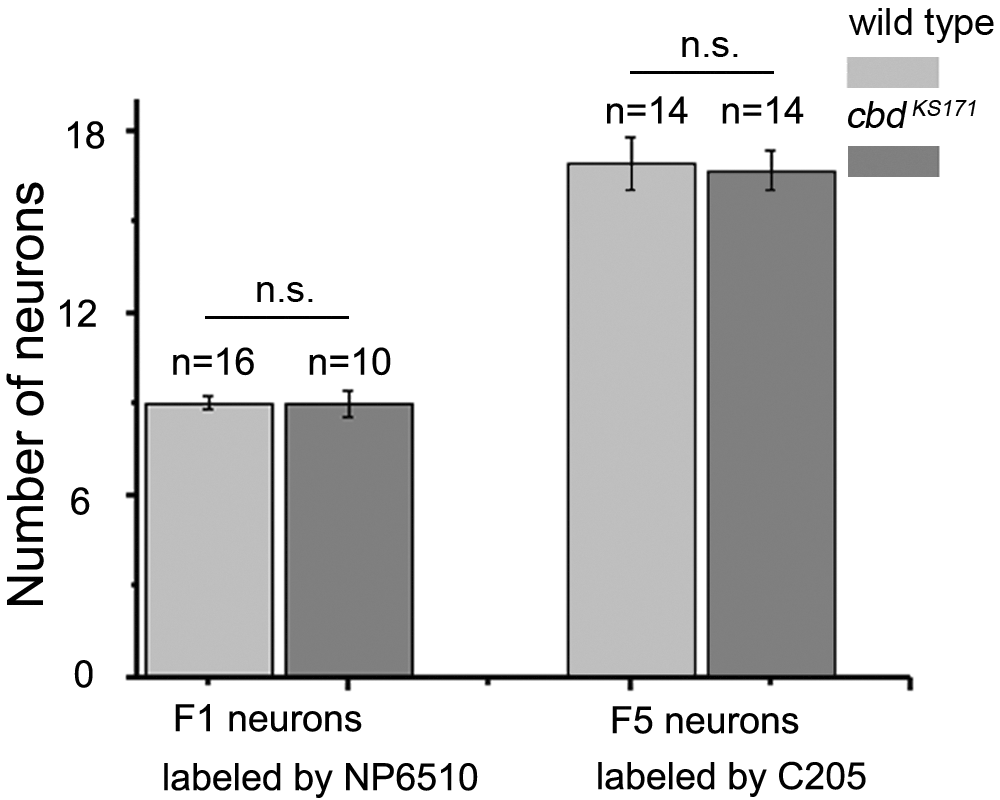

Supplement: Figure S4 — Average numbers of F1 and F5 neurons in control flies ( C205 -Gal4>UAS-GFP and NP6510 -Gal4>UAS-GFP) and cbd KS171 mutant flies. No significant difference of neuron numbers was observed between control flies (light grey) and cbd KS171 (dark grey), either for NP6510-Gal4 labeled F1 neurons (left) or for C205-Gal4 labeled F5 neurons (right). Two sample t-test, error bars represent the s.e.m.; n.s., not significant. (TIF) [file pone.0057129.s004.tif]

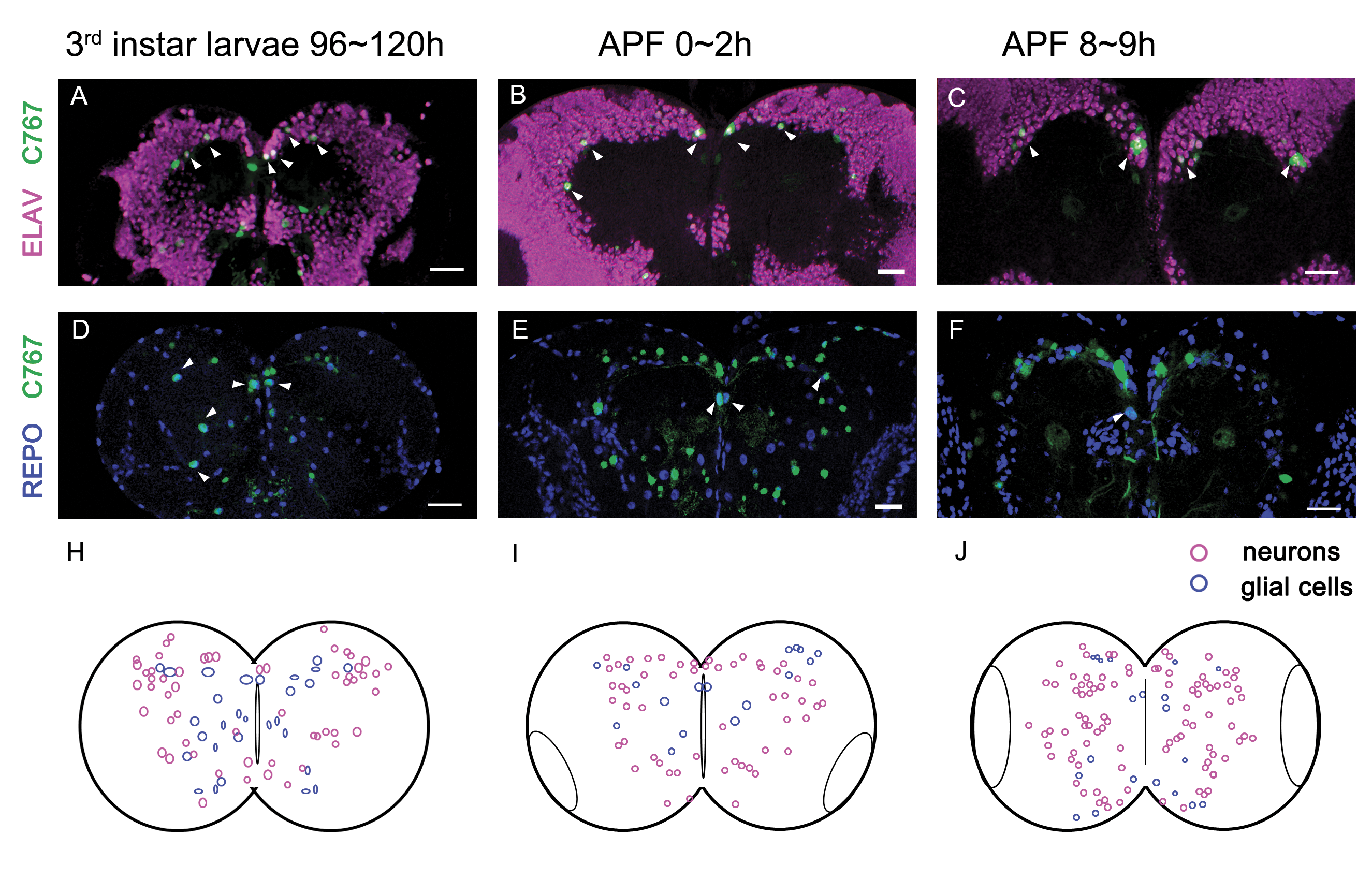

Supplement: Figure S5 — C767 -Gal4 labels both neurons and glial cells from the 3rd instar larval to early pupal stage. For easy illustration, multiple middle z-axis slices were stacked. GFP-labeled cell bodies (green) driven by C767-Gal4 co-localized with neurons (arrowheads) with a neural specific marker, ELAV, stained by anti-ELAV antibody (magenta) in 3rd instar larval brain (A), pupal brain 0∼2 h APF (B), and pupal brain 8∼9 h APF (C). GFP-labeled cell bodies driven by C767-Gal4 co-localized with glial cells (arrowheads) with a glial specific marker, REPO, stained by anti-REPO antibody (blue) in 3rd instar larval brain (D), pupal brain 0∼2 h APF (E), and pupal brain 8∼9 h APF (F). Schematics of distributions of neurons and glial cells in whole brains of 3rd instar larva (G), pupa 0∼2 h APF (H), and pupa 8∼9 h APF (I). Scale bars, 25 µm. (TIF) [file pone.0057129.s005.tif]
